# Understanding cross-data dynamics of individual and social/environmental factors through a public health lens: explainable machine learning approaches

**DOI:** 10.3389/fpubh.2023.1257861

**Published:** 2023-10-26

**Authors:** Siwoo Jeong, Sung Bum Yun, Soon Yong Park, Sungchul Mun

**Affiliations:** ^1^Convergence Institute of Human Data Technology, Jeonju University, Jeonju, Republic of Korea; ^2^Department of Sports Rehabilitation Medicine, Kyungil University, Gyeongsan, Republic of Korea; ^3^Urban Strategy Research Division, Seoul Institute of Technology, Seoul, Republic of Korea; ^4^Department of Industrial Engineering, Jeonju University, Jeonju, Republic of Korea

**Keywords:** obesity, machine learning, SHAP, GWLASSO, influential factors

## Abstract

**Introduction:**

The rising prevalence of obesity has become a public health concern, requiring efficient and comprehensive prevention strategies.

**Methods:**

This study innovatively investigated the combined influence of individual and social/environmental factors on obesity within the urban landscape of Seoul, by employing advanced machine learning approaches. We collected ‘Community Health Surveys’ and credit card usage data to represent individual factors. In parallel, we utilized ‘Seoul Open Data’ to encapsulate social/environmental factors contributing to obesity. A Random Forest model was used to predict obesity based on individual factors. The model was further subjected to Shapley Additive Explanations (SHAP) algorithms to determine each factor’s relative importance in obesity prediction. For social/environmental factors, we used the Geographically Weighted Least Absolute Shrinkage and Selection Operator (GWLASSO) to calculate the regression coefficients.

**Results:**

The Random Forest model predicted obesity with an accuracy of >90%. The SHAP revealed diverse influential individual obesity-related factors in each Gu district, although ‘self-awareness of obesity’, ‘weight control experience’, and ‘high blood pressure experience’ were among the top five influential factors across all Gu districts. The GWLASSO indicated variations in regression coefficients between social/environmental factors across different districts.

**Conclusion:**

Our findings provide valuable insights for designing targeted obesity prevention programs that integrate different individual and social/environmental factors within the context of urban design, even within the same city. This study enhances the efficient development and application of explainable machine learning in devising urban health strategies. We recommend that each autonomous district consider these differential influential factors in designing their budget plans to tackle obesity effectively.

## 1. Introduction

The global prevalence of obesity has seen a significant increase over the past decades, with the World Health Organization reporting that the obese population has tripled since 1975, and exceeded 340 million children and adolescents in 2016 (1). Particularly in South Korea, where up to 40% of the population is affected by obesity (2), there have been substantial economic and individual impacts, stemming from increased healthcare costs and various comorbidities such as cardiovascular risk (3, 4) and diabetes (5). Given this escalating trend and its profound health and economic implications, there is a need for effective strategies to manage and mitigate the escalating obesity epidemic.

Medical and pharmaceutical interventions for obesity have proven effective in curbing its prevalence (6–8). These interventions encompass a range of treatments, from weight loss medications that modulate appetite (9–12) or reduce fat absorption (13–16) to more invasive procedures such as bariatric surgeries (17–20). While they offer substantial benefits, they are not without drawbacks. For instance, weight loss medications can result in adverse effects including gastrointestinal disturbances (21, 22) and cardiovascular risk (10, 23, 24). Moreover, these medical solutions, despite being critical for some patients, predominantly address the symptoms without targeting the fundamental causes of obesity (25–27). This often results in the neglect of underlying societal and behavioral factors. Therefore, there is a demand for alternative, more comprehensive solutions to managing obesity.

Obesity prevention programs aim to address the multifaceted root causes of the condition while minimizing associated side effects. Root causes involve individual factors such as dietary habits, physical activity, genetic predisposition, and lifestyle choices. Concurrently, social/environmental factors including access to healthy food options, availability of recreational facilities, socioeconomic status, and urban planning play crucial roles (28–35). Based on their primary focus, prevention programs for obesity can be broadly classified as individual-based or social/environmental-based categories (36). Whereas individual-based programs focus on nutritional behavior, physical activity, and media consumption (37–43), social/environmental-based programs target factors such as neighborhood socioeconomic status, accessibility to green parks, and public transportation frequency (36, 44). Neither approach is universally effective, as individual-based programs might not reach certain at-risk groups (36), and social/environmental-based interventions could potentially be less effective overall (45). Therefore, it is essential for successful obesity prevention to integrate individual and social factors, exploring their simultaneous influence on obesity.

Machine learning approaches are well-suited for developing obesity-related models due to their ability to handle numerous multidomain influencing factors and identify complex relationships (46–48). However, machine-learning approaches have limitations in determining obesity-related factors. First, it is not interpretable for machine learning models due to its black-box properties. Although machine learning classification models distinguish obesity with high accuracy and reveal obesity risk factors (49–51), the extent to which each variable contributes to obesity is not provided. When developing an obesity prevention program or policy, the most influential factors should be considered to ensure its efficiency and effectiveness. However, traditional machine learning models are not explainable, which prevents the selection of the most influential obesity risk factors. Second, public social/environmental factors are rarely labeled as obesity. Since social/environmental data are usually gathered by using public statistical data or open sources and not by individual surveys, these factors cannot be directly connected to dependent variables such as weight, body mass index (BMI), or obesity. This limitation prevents the development of classification models for social/environmental factors.

To circumvent these limitations, this study used Shapley additive explanations (SHAP), an explainable machine-learning model (52, 53), to determine how obesity risk factors influence obesity. After the implementation of the machine learning classification models, the model can be applied to SHAP, which identifies the contribution of each factor to obesity. Additionally, geographically weighted least absolute shrinkage and selection operator (GWLASSO) was used to reveal how social/environmental factors influenced obesity by considering spatial relationships (54). GWLASSO may improve the reliability of the results by deriving the factors influencing individuals’ activity range, including neighboring cities or districts. The integration of SHAP and GWLASSO in this study provides a comprehensive and spatially nuanced understanding of the multifaceted factors contributing to obesity, enhancing our ability to develop targeted and effective interventions.

Despite the increasing volume of research on obesity and its risk factors worldwide (55–57), a comprehensive understanding of the intricate interplay between individual and social/environmental factors within a specific population or region remains elusive. Bohnert et al. investigated childhood overweight and obesity rates in the United States, emphasizing their persistence into adulthood, as well as associated healthcare costs and health issues (58). They explored how principles from developmental psychopathology, including multilevel modeling, can enhance the understanding of obesity risk examining developmental pathways and complex processes. Their findings offered a novel perspective for more effective intervention and prevention efforts in addressing the obesity epidemic. Zare et al. also delved into the intricate interplay of multiple factors, including income levels, racial and ethnic differences, and employed multiple modeling approaches to understand their relationship with obesity among U.S. adult men (59). By utilizing data spanning from 1999 to 2016, income was categorized, and income inequality was measured through the Gini coefficient. Their findings highlighted a noteworthy association between income and obesity, particularly among Non-Hispanic White and Non-Hispanic Black populations. This underscored the importance of developing race-specific strategies to address income inequality within the context of obesity prevention, while using insights gained from these diverse modeling techniques. Previous studies, however, including those utilizing multilevel modeling in the United States, have investigated the impacts of individual behaviors and neighborhood-level factors on obesity prevalence and offered valuable insights into the multifaceted nature of obesity risk factors. They have also highlighted the interrelation between individual behaviors and neighborhood-level attributes (60–62). However, these studies often did not consider environmental factors such as urban planning or availability of public spaces for physical activity, which are crucial for a comprehensive exploration of obesity risk factors. Moreover, the generalization of these findings to regions distinct in their cultural, social, and urban environments can be problematic due to inherent regional discrepancies. This underscores the importance of research tailored to the distinct characteristics and needs of each specific region.

While there has been a sharp increase in obesity in South Korea, there have been few studies that investigated both individual and other social level factors of obesity within the same region. Considering the individualistic nature of Korean society, where interactions with the neighborhood are minimal, there is a need for integrated research examining both social/environmental and individual factors. Considering the specific societal dynamics of South Korea, a detailed exploration of the interplay between social/environmental and individual factors is essential. To address this, our study utilized advanced machine learning approaches, such as SHAP and GWLASSO, to investigate the factors impacting obesity in Seoul, South Korea, focusing on both individual and social/environmental factors. Therefore, this study aimed to (1) identify the influential factors related to obesity from both individual and social/environmental perspectives for each Gu district of Seoul using machine learning models, including SHAP and GWLASSO, and (2) assessed the relative contributions of these factors to obesity prevalence. The results of this study could provide critical insights for the development of comprehensive and effective obesity prevention strategies and inform urban design decisions that promote healthier living.

## 2. Methods

**Figure 1:**
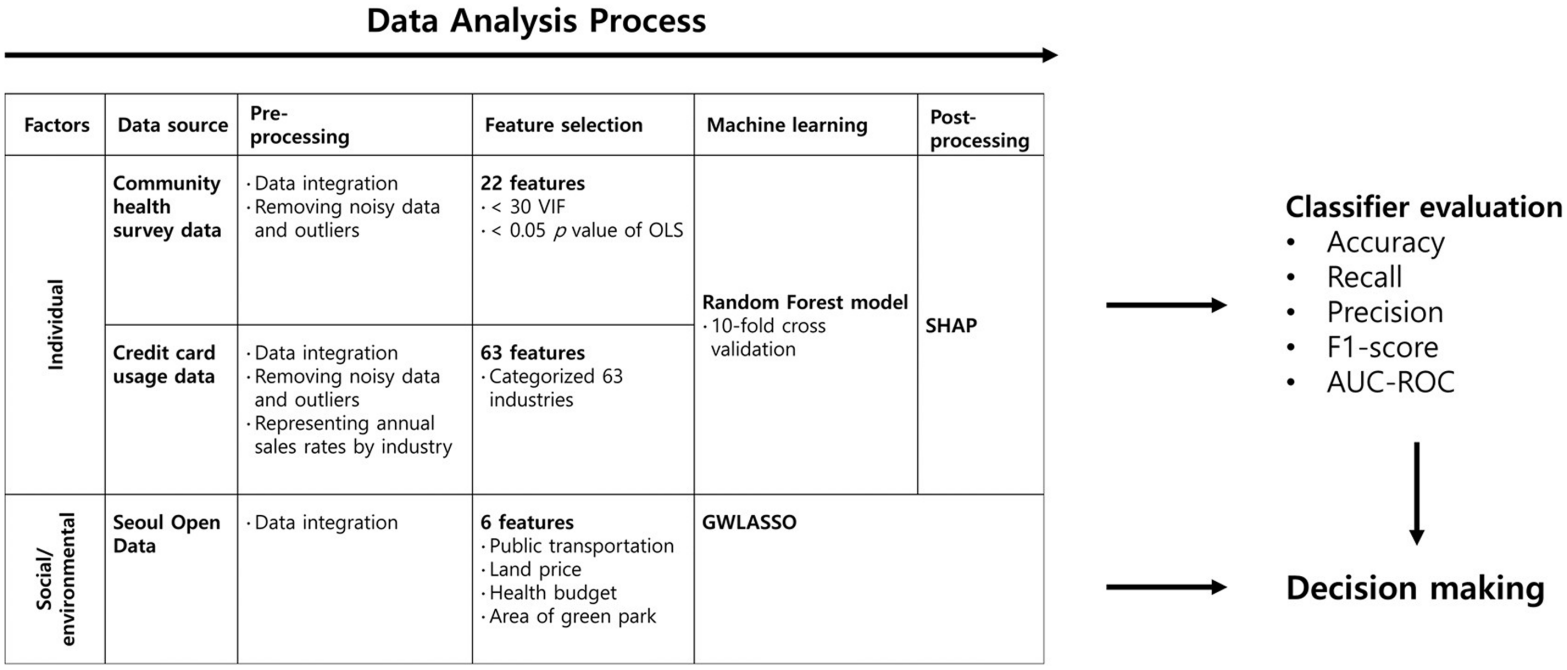
Methodology of predicting obesity risk and deriving the influential factors of obesity.

Three different public and open datasets were collected from Seoul City and the Korean Ministry of Health and Welfare (MOHW). Three diverse public and open datasets were collated from Seoul City and the MOHW, specifically community health survey data, credit card usage data, and Seoul Open Data. These datasets underwent a preprocessing stage to mitigate the impact of noisy data and outliers and were subsequently categorized at the Gu-administrative district level in Seoul City. For the community health survey data, entries with ‘no response’ were systemically excluded, ensuring the removal of the corresponding individual’s data from analysis. The credit card data, refined and provided by Shinhan Card Company (South Korea), were assessed to be devoid of noise, representing reliable average values for the respective areas. For Seoul City’s open data, any erroneous values were replaced with the overall district average to ensure data integrity and reliability. To derive the individual influential factors leading to obesity in each Gu district, contributing features were selected and applied to a machine learning model (Random Forest model). The trained model was validated using 10-fold cross-validation and interpreted using SHAP, which extracted the values of the contribution of each feature to obesity. Regarding social/environmental factors, GWLASSO was used to determine the coefficient in the regression model between obesity and social/environmental factors (Figure 1). More details on the data collection, feature selection, and processes for determining the best-performing model in the test sets are described in the following subsections.

### 2.1. Data collection

In the current study, community health survey data, credit card usage data, and Seoul Open Data were used to identify the influential factors leading to obesity. These data were acquired from the MOHW and Seoul City with authorization. The credit card data used in this study were provided by Shinhan Card Company. These data were released for research purposes after undergoing a reprocessing by Shinhan Card Company to ensure confidentiality and compliance with data protection regulations. Before releasing the data, Shinhan Card obtained consent from cardholders by having them check a third-party information provision consent form when issuing the card, which legally authorizes the use of such data for research purposes. Community health survey data and credit card usage data were utilized for individual data, and Seoul Open Data were utilized for social/environmental data (Table 1). We calculated the BMI using the available height and weight data. Based on the World Health Organization’s classification, participants were then categorized into obesity and non-obesity groups using a BMI of 25 as the dividing line. Subsequently, analyzes were conducted incorporating both the obesity and non-obesity groups. Random oversampling was conducted to match the number of obesity and non-obesity data points.

**Table 1:** Data sets used for model training.

Factor	Data source	Description	Number
Individual	Community health survey data	Raw data collected from yearly community health survey data from 2017 to 2019	114,789
	Credit card usage data	Daily credit card sales information	1,512,232
Social/ environmental	Seoul Open Data	Comprises the area of green park, public transportation information (bus and subway), land price, bicycle utilization, health budget, and spatial information on each Gu-district in Seoul city	Green Park: 131 Bus: 10,985 Subway: 645 Land price: 909,496 Bicycle: 12,462,884 Health budge: 75

#### 2.1.1. Community health survey data

The target population of the community health survey data was adults aged over 19 years living in South Korea at the time of the survey (16^th^ August, annually). Surveys were divided into household and individual levels and collected from 2017 to 2019. A household survey was conducted to obtain data on household income, type of house, type of household, and basic livelihood security recipient. The health measurement data in this study were obtained from self-reported responses of the participants. Special attention was given to providing clear instructions to participants, enhancing the accuracy and reliability of the collected data. Self-reported data covered a range of factors, including personal measurements (blood pressure, height, and body weight), health-related information (obesity, weight, smoking, drinking, safety awareness, physical utilization, diet, oral health, and mental health), medical screenings (influenza vaccination, health checkups, and cancer screening), morbidities (chronic disease diagnosis experience and management level of major diseases), medical care use (number of visits in a year), accidents (experiences of major accidents), activity restrictions, and quality of life (subjective health level, EQ-5D scores). The study also considered factors related to the use of health institutions, socio-physical environments (duration of residence in urban areas or specific districts, which is critical due to substantial urban-rural disparities in living conditions and lifestyles), personal hygiene, women’s health (pregnancy status), education, and economic activities (occupation, education, marital status) as shown in Table 1. The ‘community health survey data’ used in this study included a broad spectrum of health-related variables. For the purpose of our study, we specifically selected a subset of these variables, mainly those relevant to obesity and its associated factors. The selected survey data for individuals living in Seoul City were categorized into the Gu-administrative district level and the lower level of the city. Categorized data were used along with other datasets.

#### 2.1.2. Credit card usage data

Credit card usage data were used to identify the expenditure patterns of individuals living in each district of Seoul (Table 1). Credit card data included daily average sales information and, to use this data along with other datasets, the spatial unit was changed to that in other datasets. The sales data were aggregated at the Gu-administrative district level and the annual average values were calculated.

### 2.2. Data processing and analysis

**Figure 2:**
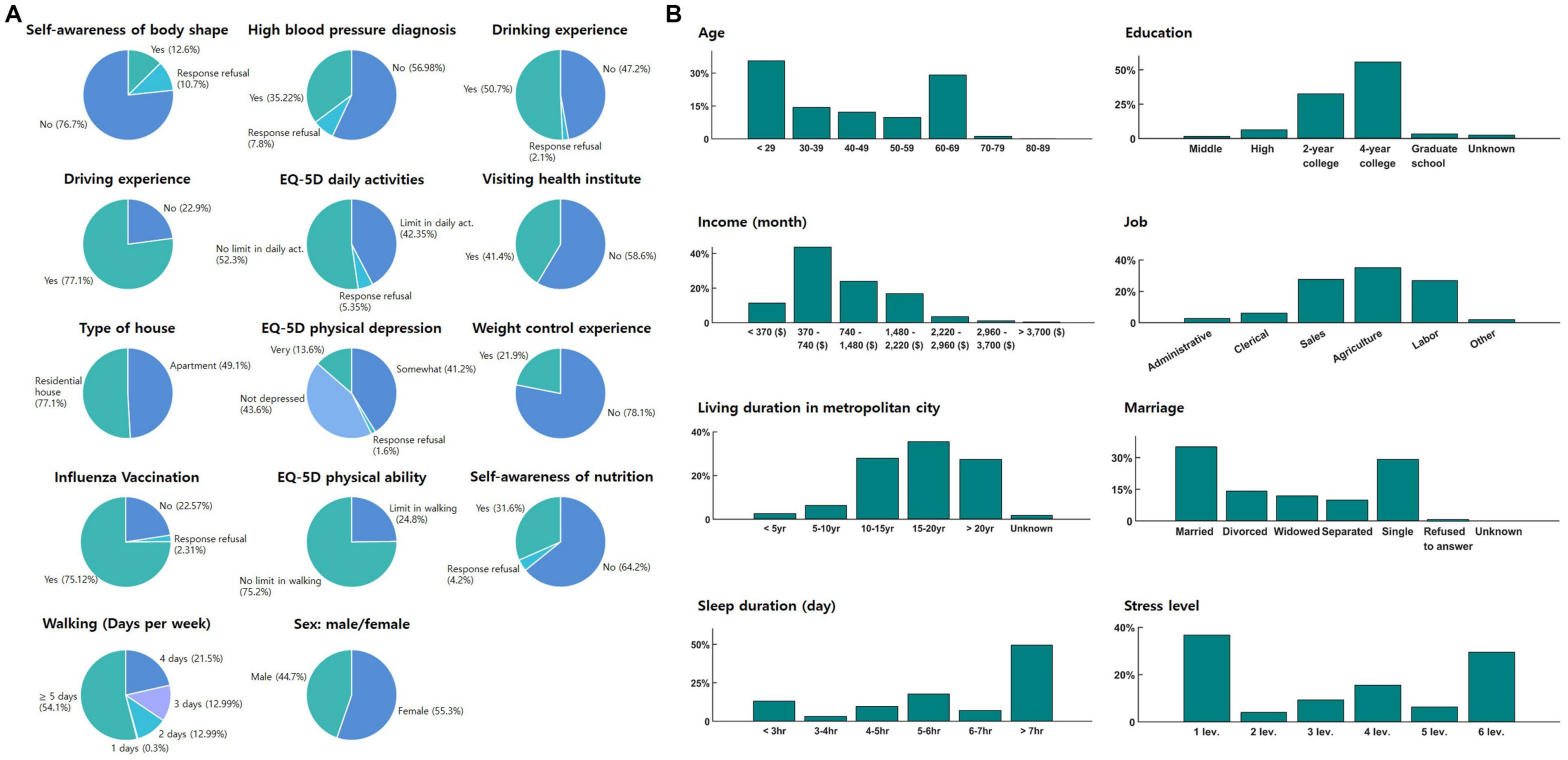
Descriptive statistics diagrams of selected features from ‘Community Health Survey Data’: **(A)** pie chart and **(B)** bar graph. **(A)** The pie chart provides a detailed representation of the distribution of various categorical variables such as ‘Self-awareness of body shape’, ‘High blood pressure diagnosis’, ‘Drinking experience’, ‘Driving experience’, ‘EQ-5D indexes’, ‘Visiting health institute’, ‘Type of house’, ‘Weight control experience’, ‘Influenza vaccination’, ‘Self-awareness of nutrition’, ‘Walking’, and ‘Sex’, categorized as ‘male’ or ‘female’. **(B)** The bar graph illustrates the distribution of variables like ‘Age’, ‘Education’, ‘Income’, ‘Job’, ‘Living duration in metropolitan city’, ‘Marriage’, ‘Sleep duration’, and ‘Stress level’.

Community health survey data were categorized into individual factors. Raw data were preprocessed to integrate them and eliminate redundant data and outliers. A total of 32 common responses suitable for analyzing obesity-related factors were extracted, excluding sub-questions and questions unrelated to obesity (Table 2; Figure 2). Credit card usage data were categorized into individual factors and converted to the annual sales rate by industry (Table 3). Social/environmental factors were obtained from Seoul Open Data, which was preprocessed. Since each Gu-administrative district had a different area and population size, all data categorized by district were normalized by dividing data by the population of each district, resulting in data per person for each district.

**Table 2:** Features for individual factors from ‘Community health survey data.

No.	Feature	Selection
1	Sex: male/female	O
2	Type of house	O
3	Type of household	X
4	Household income	O
5	Smoking experience	X
6	Electronic-cigarettes experience	X
7	Drinking experience	O
8	Driving experience	O
9	Bicycle experience	X
10	Walking duration	O
11	Self-awareness of nutrition	O
12	Self-awareness of body shape	O
13	Regular dentals check up	X
14	Sleep duration	O
15	Stress level	O
16	Influenza vaccination	O
17	High blood pressure diagnosis	O
18	Diabetes diagnosis	X
19	EQ_5D physical ability	O
20	EQ_5D self-discipline	X
21	EQ_5D daily activities	O
22	EQ_5D pain	X
23	EQ_5D depression	O
24	Visiting health institute	O
25	Living duration in metropolitan city	O
26	Living duration in city	X
27	Economic activity experience	X
28	Job	O
29	Education	O
30	Marriage	O
31	Age	O
32	Weight control experience	O

EQ_5D: five representative factors of health and life quality.

**Table 3:** Categories of credit card data.

Main category (13)	Middle category (30)	Sub category (63)
Retail/Restaurant	Korean food	Korean food
	Japanese/Chinese/Western food	Japanese, Chinese, Western food
	Bakery/coffee/fast-food	Bakery, coffee, fast-food
	Others	Others
	Entertainment	Karaoke, other entertainments, bar
Product distribution	Department store	Department store
	Discount store/supermarket	Discount store, supermarket, household goods
	Convenience store	Convenience store
	Others	Others
Food/beverage	Food/beverage	Meat, vegetable, others
Clothing/accessories	Clothing	Clothing
	Fashion accessories	Fashion accessories, watch, glasses
Sports/culture/leisure	Sports/culture/leisure	Indoor golf/fitness, outdoor golf/ski, leisure facility, movie/concert, sports facility, recreation, bookstore
	Sports/culture/leisure goods	Sports goods, culture goods, flower
Travel/residence	residence	Hotel/condo, others
	travel	Travel company, duty-free store
Beauty	hair	Hair shop, hair-related service
	cosmetics	cosmetics
Home life/service	Service	Laundry service, daily activity service, business service
	Interior design	Interior design
Education/academy	Education	Private reading room, academy
	Childhood education	Childhood education
	Education goods	Education goods
Medical service	hospital	General hospital, local hospital, dental clinic, Korean medicine
	Pharmacy	Pharmacy
	others	others
Electronics/furniture	Electronics/furniture	Electronics, furniture, others
Car	Car sales	Car sales
	Car service/car goods	Car-related service, car goods
Fuel	Oil	Oil, LPG

#### 2.2.1. Feature selection

Before feature selection, we considered a range of individual and social/environmental risk factors based on the existing literature on obesity (36–42, 44, 45). For individual factors, we included variables such as age, sex: male/female, occupation, education level, dietary habits, smoking, physical activity, sleep duration, stress level, blood pressure, diabetes, marriage, influenza vaccination, self-awareness of nutrition and body shape, and weight control experience. For social/environmental factors, we considered variables such as the availability of public transportation, green spaces, and recreational facilities, as well as socioeconomic indicators such as official land prices and individual health budgets for residents. The rationale for including these factors was based on their potential impact on obesity prevalence in Seoul City and their relevance in previous research.

To improve model performance, features were selected using the following statistical methods: The variance inflation factor (VIF) was used for the 32 respondents in the community health survey to evaluate multicollinearity. Although a VIF value greater than 10 typically indicates multicollinearity (63), employing a threshold of 10 VIF was deemed unsuitable in this study because it led to the removal of critical factors. Kim suggested that VIF values between 10 and 30 may indicate the presence of multicollinearity, but it is not strong (64). Therefore, through trial and error, we determined that a 30 VIF threshold effectively reduced multicollinearity without excluding crucial factors from the analysis. Additionally, ordinary least squares regression was performed to obtain a simple linear regression for the dataset. Obesity was set as the dependent variable. Variables showing a *value of p* greater than 0.05 were excluded. Ultimately, 22 factors from the ‘Community Health Survey Data’ were selected as individual features applied to the machine learning model (Table 2). From the ‘Credit card use data’, 63 categorized factors were also used as individual features to develop obesity-related machine learning models (Table 3). Selected social and environmental features included factors related to the area of green parks, public transportation, bicycles, and official land prices.

#### 2.2.2. Machine learning models

For classification purposes, four different machine-learning models were applied: Logistic Regression, Random Forest, XGBoost, and Gradient Boosting. All classification models were trained and validated using community health survey data. Specifically, 10-fold cross-validation was employed. The results showed that the random forest model outperformed the other classification models in this dataset. Therefore, the random forest model was selected to classify obesity using the selected 22 features from Community health survey data and Credit card use data to derive the influential individual features leading to obesity.

The random forest algorithm is a decision-tree algorithm that uses an ensemble learning mechanism to create classification or regression models (65). Similar to the decision tree algorithm, the random forest algorithm requires target and input variables, where the target variable is a predefined class, such as a category or a continuous value. The target variable was used as the basis for the tree model analysis. The input variables were “pool of data” from which the random forest algorithm could extract factors that influence the pre-defined class or continuous value. The major difference between the original decision tree and the random forest is that the random forest algorithm creates multiple trees by randomly selecting variables from the given input variables through the process of bagging. This procedure reduces overfitting, which is a critical disadvantage of the original decision tree algorithm. In this study, the random number of trees to be created was set to 5,000 to ensure the full random usage of all data.

#### 2.2.3. SHAP algorithm (Shapley analysis)

The results of machine learning algorithms are not interpretable due to their “black box” properties. To derive obesity-related factors from the machine learning results, the SHAP algorithm was used to provide the influential weight of each feature for prediction (66). The influential weight represents the extent to which individual features contribute to the prediction.

**Figure 3:**
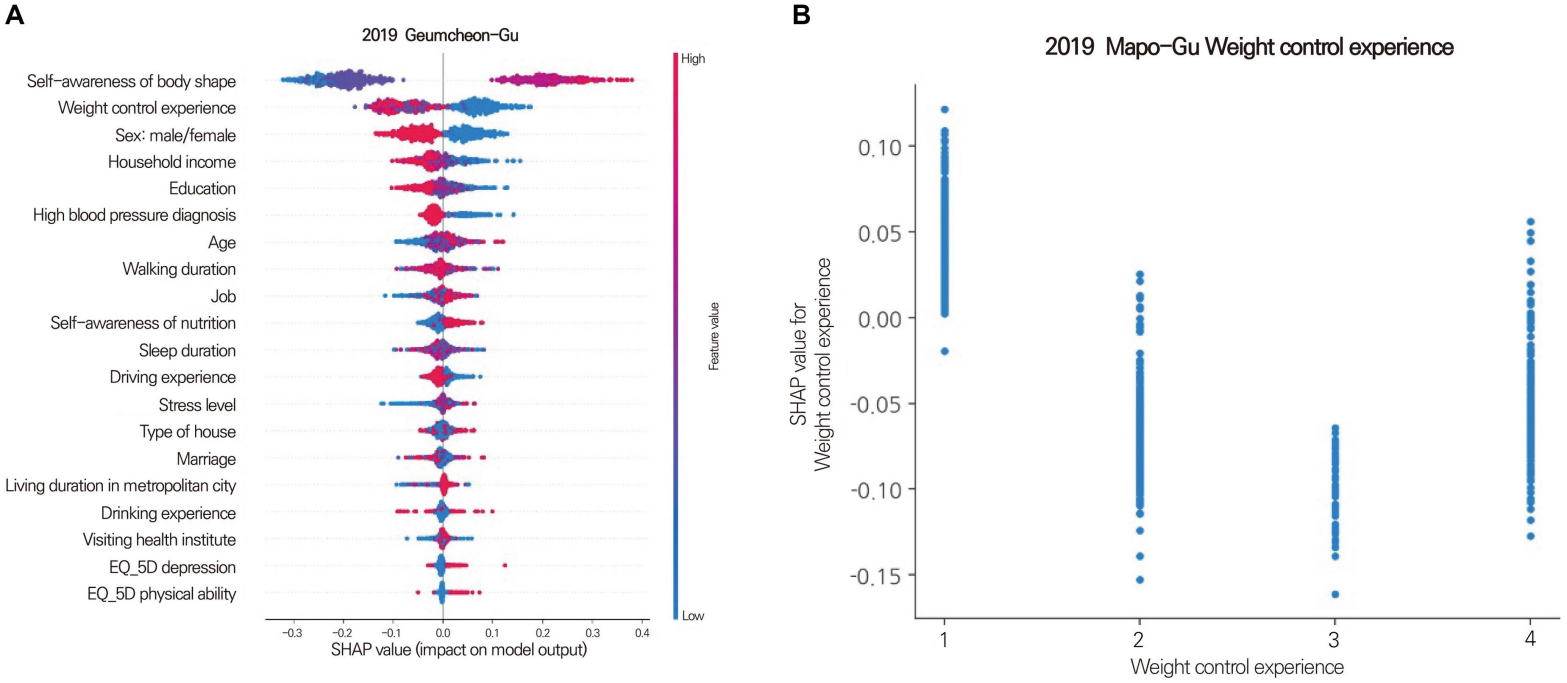
Shapley summary plot **(A)** for Geumcheon-Gu and dependence plots **(B)** for Mapo-Gu. **(A)** The SHAP value (x-axis) represents the impact on obesity. Positive values indicate contributions to obesity, while negative values indicate contributions to non-obesity. The absolute value signifies the magnitude of the contribution, with a SHAP value of 0 meaning no contribution. Features are sorted in descending order of importance from top to bottom. Colors represent the response value of the feature, with red indicating high values and blue indicating low values. For instance, red in the age feature represents older age, while blue signifies younger age. A clear separation of colors for each feature based on a SHAP value of 0 suggests that the feature is an influential factor. **(B)** The Shapley dependence plot illustrates how each response contributes to the SHAP value. The x-axis represents the responses to the question about weight control experience, with higher numbers indicating more frequent experiences of weight control. The y-axis represents the SHAP value for weight control experience. In Mapo-Gu, individuals with less experience in weight control have a higher likelihood of developing obesity.

There are two approaches to the SHAP algorithm: KernelSHAP and TreeSHAP (67). In this study, treeSHAP was used because of its faster processing speed than kernelSHAP. The SHAP results were presented as SHAP summary and dependence plots. The SHAP summary plot showed the extent to which individual features influenced the prediction with the combined value of feature importance and feature effects (Figure 3A). A SHAP dependence plot was used to investigate discriminant features of the SHAP summary plot results. This plot showed how the responses to each survey question were related to obesity (Figure 3B).

#### 2.2.4. GWLASSO

The shrinkage method in statistical learning is used to reduce the effects of sampling variation. The least absolute shrinkage and selection operator (LASSO) is widely used for variable selection. GWLASSO is a modified LASSO that alleviates the collinearity effect among explanatory factors by adding geographical weights, which allows the implementation of variable selection with spatial information (68). In this study, GWLASSO was used to identify the social/environmental influencing factors leading to obesity with potential spatial relationship patterns among Gu districts using the Euclidean distance between each district (54). The GWLASSO equation is as follows:


$$y_{i}=\beta_{0}\left(u_{i},v_{i}\right)-\overset{p}{\underset{k=1}{\sum }}x_{ik}\beta_{k}\left(u_{i},v_{i}\right)+\varepsilon_{i}$$

where $y_{i}$ and $x_{ik}$ represent the rate of obesity in *i* Gu district and *k* social/environmental factor in *i* Gu district, respectively. $\beta_{k}$ represents the estimated regression coefficient of the *k* factor. $\varepsilon_{i}$ represents the residual at *i* Gu district, and $u_{i}$ and $v_{i}$ indicate the position in longitude and latitude, respectively. The GWLASSO coefficient estimates were defined as:


$${\hat{\beta }}_{GWL}=\mathrm{argmin}\left\{\begin{array}{l} \overset{n}{\underset{i=1}{\sum }}{\left(y_{i}-\beta_{0}\left(u_{i},v_{i}\right)-\overset{p}{\underset{k=1}{\sum }}x_{ik}\beta_{k}\left(u_{i},v_{i}\right)\right)}^{2}\\ +\lambda \overset{p}{\underset{k=1}{\sum }}|\beta_{k}\left(u_{i},v_{i}\right)| \end{array}\right\}$$

The optimal λ for each Gu district was selected within the range of 0.001 to 0.03.

#### 2.2.5. Evaluation of classification performance

The developed machine learning model, using the random forest algorithm for classification, was evaluated by comparing the predicted labels with the true labels. The performance of the model was defined based on accuracy, precision, recall, f1-score, and AUC.


$$\mathrm{Accuracy}=\frac{TP+TN}{TP+FP+TN+FN}\times 100\%$$

$$\mathrm{Precision}=\frac{TP}{TP+FP}\times 100\%$$

$$\mathrm{Recall}=\frac{TP}{TP+FN}\times 100\%$$

$$F1-\mathrm{score}=\frac{\mathrm{Sensitivity}\times \mathrm{Precision}}{\mathrm{Sensitivity}+\mathrm{Specificity}}\times 2$$

True positive (TP) referred to the number of participants correctly labeled as obese, true negative (TN) referred to the number of participants correctly labeled as non-obese, false positive (FP) referred to the number of participants incorrectly labeled as obese, and false negative (FN) referred to the number of participants incorrectly labeled as non-obese. The accuracy and precision indicated how close the predicted obesity was to the true obesity and the quality of the positive obesity label, respectively. The recall indicated the ability of the model to recognize obesity. The F1-score represented the harmonic mean of precision and recall.

## 3. Results

### 3.1. The rate of obesity

**Figure 4:**
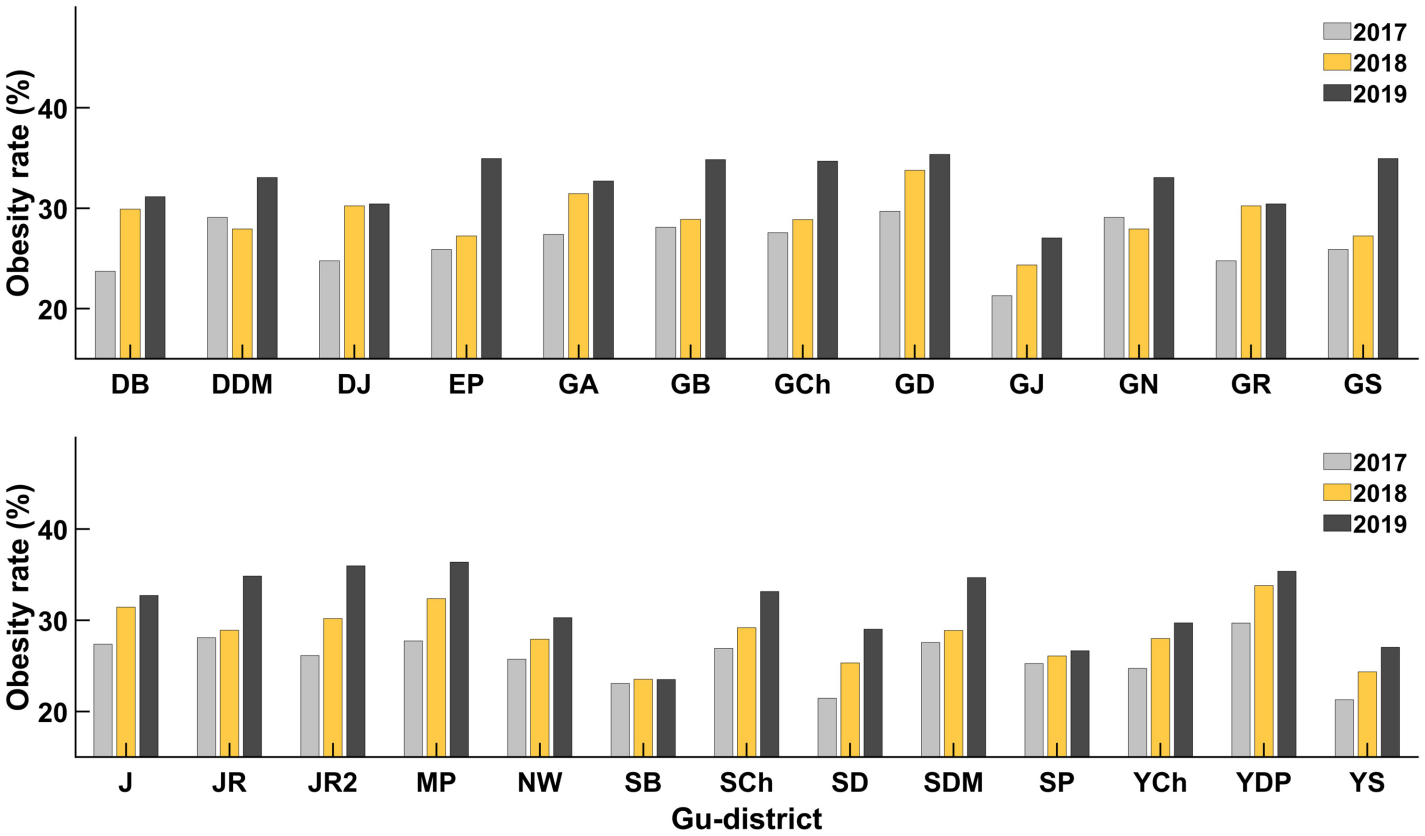
Obesity rates for all gu-districts from 2017 to 2019. The rate of obesity increased from 2017 (gray) to 2019 (black) in all gu-districts. In every gu-district, the obesity rate in 2019 was higher than in 2017 (DB, Dobong-gu; DDM, Dongdaemoon-gu; DJ, Dongjak-gu; EP, Eunpyung-gu; GA, Gwanak-gu; GB, Gangbuk-gu; GCh, Geumcheon-gu; GD, Gangdong-gu; GJ, Gwangjin-gu; GN, Gangnam-gu; GR, Guro-gu; GS, Gangseo-gu; J, Jung-gu; JR, Jungrang-gu; JR2, Jongro-gu; MP, Mapo-gu; NW, Nowon-gu; SB, Seongbuk-gu; SCh, Seocho-gu; SD, Seongdong-gu; SDM, Seodaemoon-gu; SP, Songpa-gu; YCh, Yangcheon-gu; YDP, Youngdeungpo-gu; YS, Yongsan-gu).

**Figure 5:**
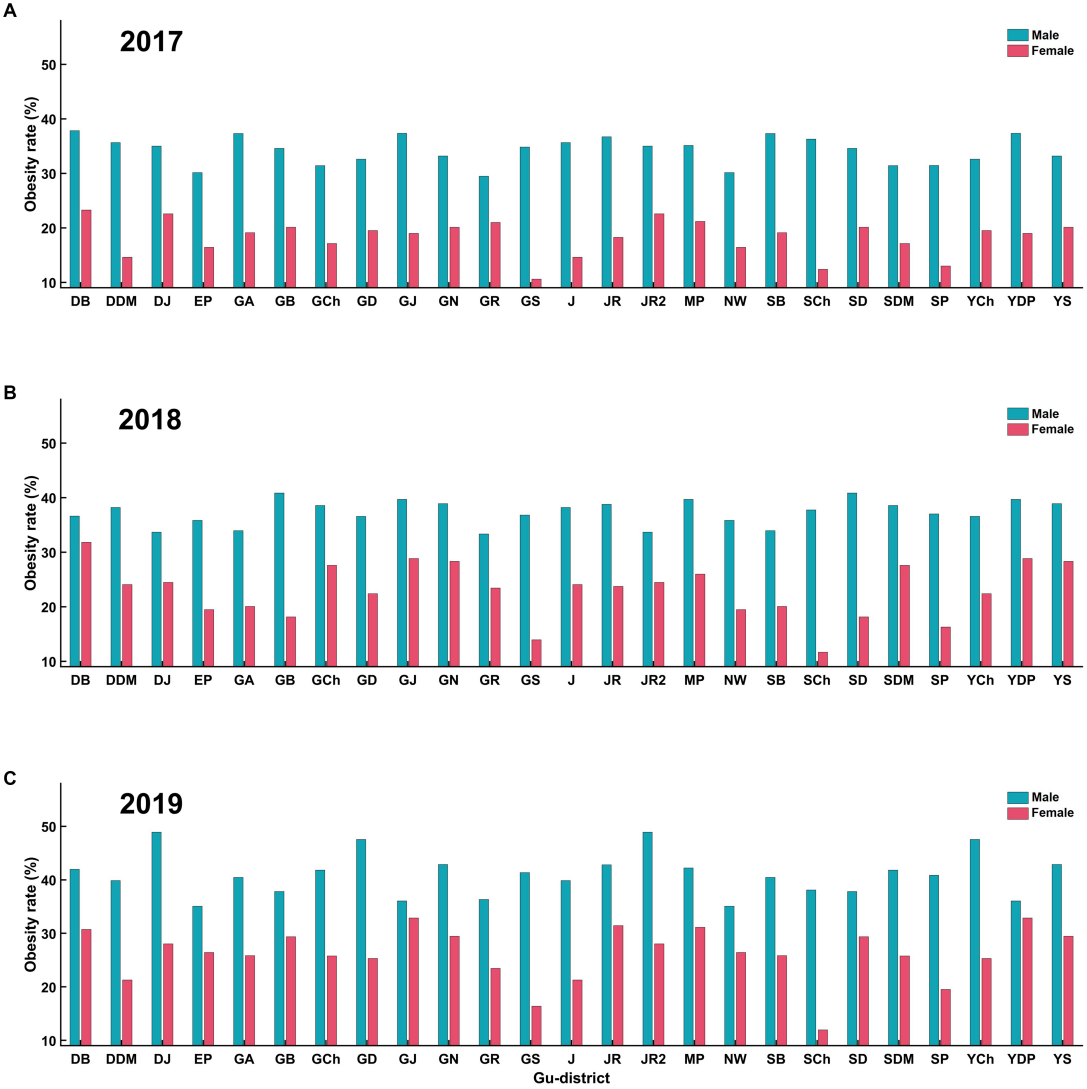
Obesity rates by sex for all gu-districts on **(A)** 2017, **(B)** 2018, and **(C)** 2019. **(A)** represents obesity rates in 2017, with males (blue) consistently having higher rates than females (red) across all gu-districts. **(B)** displays the same trend for 2018. Similarly, **(C)** represents data for 2019. Across these years, male obesity rates in all gu-districts were consistently higher than those for females.

**Figure 6:**
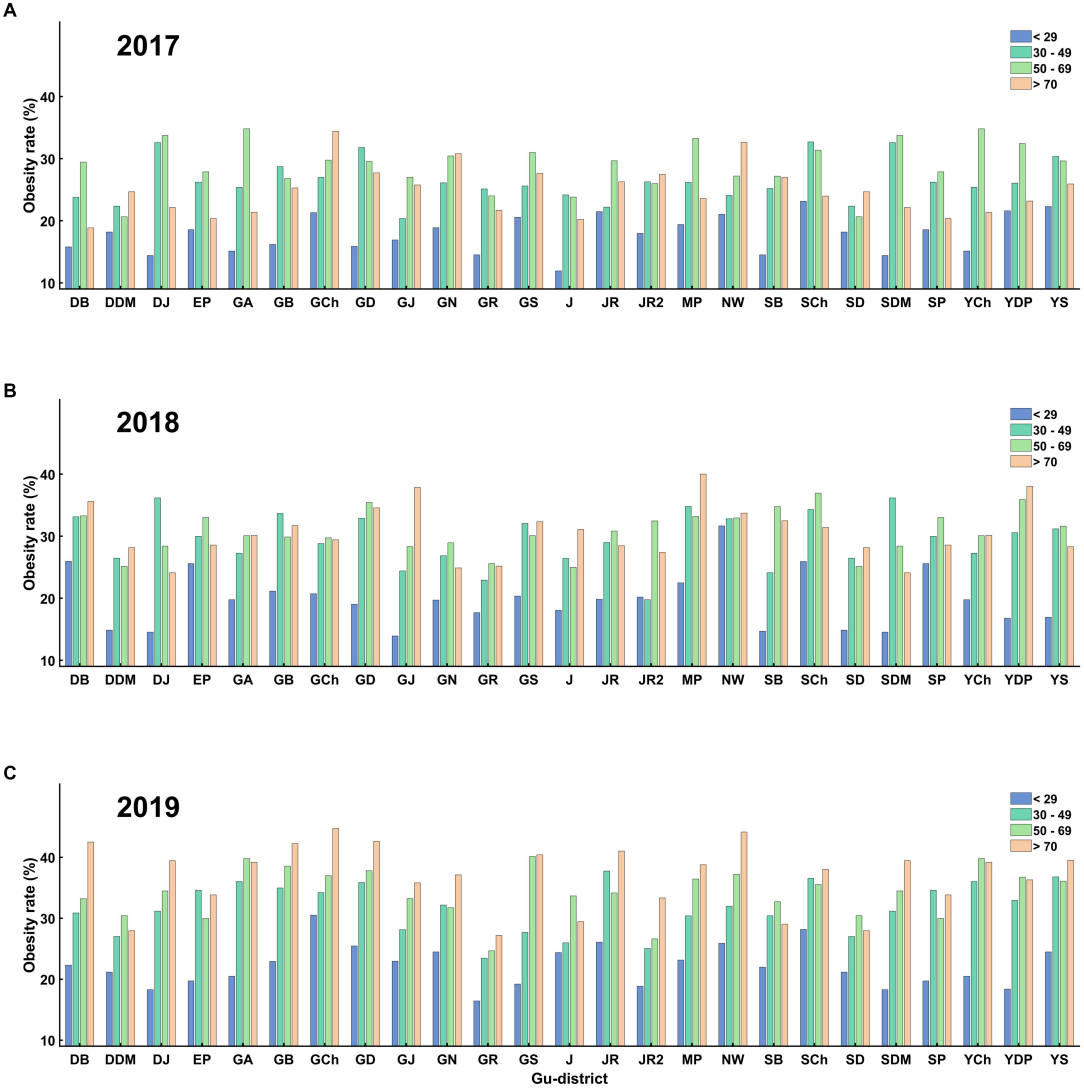
Obesity rates for different age groups across all gu-districts on **(A)** 2017, **(B)** 2018, and **(C)** 2019. **(A)** shows the obesity rates across age groups in 2017. The trend indicates that older individuals (>70 years) have a consistently higher obesity rate than younger individuals (<29 years). **(B)** displays the same trend for 2018, and **(C)** for 2019. Throughout these years, the obesity rate of older age groups was consistently higher than the younger age groups in all gu-districts.

According to the community health survey data, all Gu districts in Seoul have experienced an increased obesity rate since 2017. Gangnam-Gu (21.3%, 2017; 24.3%, 2018; 27.0%, 2019) and Seocho-Gu (23.1%, 2017; 23.5%, 2018; 23.5%, 2019) had the lowest obesity rates in 2017, 2018, and 2019. In contrast, Jungrang-Gu (29.7%, 2017; 33.9%, 2018; 35.4%, 2019) and Dobong-Gu (28.2%, 2017; 28.6%, 2018; 37.0%, 2019) had the highest obesity rates in 2017, 2018, and 2019. The difference between the lowest and highest obesity rates was approximately 10% annually (Figure 4). The rate of obesity in women (22.5 ± 5.3%) was approximately 10% higher than that in men (36.9 ± 3.9%) (Figure 5), and older adults (31.5 ± 6.7%, > 69 years of age) showed approximately 10% higher rate of obesity compared to young adults (20.2 ± 4.0%, < 29 years of age) in all districts (Figure 6).

### 3.2. Classification of obesity at each Gu district level

The classification results are presented in detail in Table 4. The random forest model showing the best classification performance was used for the classification of obesity in each Gu district of Seoul City. The pre-processed 88 individual features were applied to the random forest model. The random forest approach for distinguishing between obesity and non-obesity yielded the highest performance, with an accuracy of 96%, in the Songpa-Gu district and the lowest performance, with an accuracy of 83%, in the Dondaemoon-Gu district. The average (SD) of accuracy, precision, recall, and f1-score were 90% (3%), 95% (4%), 85 (5%), and 89% (3%), respectively (Table 4).

**Table 4:** Accuracy, precision, recall, and F1-score values of random forest model used to classify obesity.

	Accuracy	Precision	Recall	F1-score
Gangnam-Gu	0.93	0.96	0.89	0.93
Gangdong-Gu	0.91	0.98	0.84	0.90
Gangbuk-Gu	0.89	0.91	0.85	0.88
Gangseo-Gu	0.94	1.00	0.89	0.94
Gwanak-Gu	0.89	0.93	0.84	0.88
Gwangjin-Gu	0.89	0.94	0.84	0.89
Guro-Gu	0.92	0.98	0.86	0.92
Geumcheon-Gu	0.92	0.98	0.86	0.92
Nowon-Gu	0.90	0.95	0.83	0.89
Dobong-Gu	0.90	0.91	0.88	0.89
Dondaemoon-Gu	0.83	0.85	0.80	0.82
Dongjak-Gu	0.89	0.97	0.81	0.88
Mapo-Gu	0.86	0.97	0.75	0.85
Seodaemoon-Gu	0.93	0.97	0.88	0.92
Seocho-Gu	0.88	0.89	0.86	0.88
Seongdong-Gu	0.90	0.96	0.83	0.89
Seongbuk-Gu	0.90	0.91	0.88	0.89
Songpa-Gu	0.96	0.96	0.95	0.96
Yangcheon-Gu	0.87	0.88	0.85	0.86
Youngdeungpo-Gu	0.92	0.95	0.88	0.92
Yongsan-Gu	0.94	0.98	0.89	0.93
Eunpyung-Gu	0.86	0.88	0.84	0.86
Jongro-Gu	0.88	0.95	0.80	0.87
Jung-Gu	0.92	1.00	0.84	0.91
Jungrang-Gu	0.86	1.00	0.72	0.84
Total	0.90 ± 0.03	0.95 ± 0.04	0.85 ± 0.05	0.89 ± 0.03

### 3.3. SHAP algorithm to determine contributing factors of obesity

**Figure 7:**
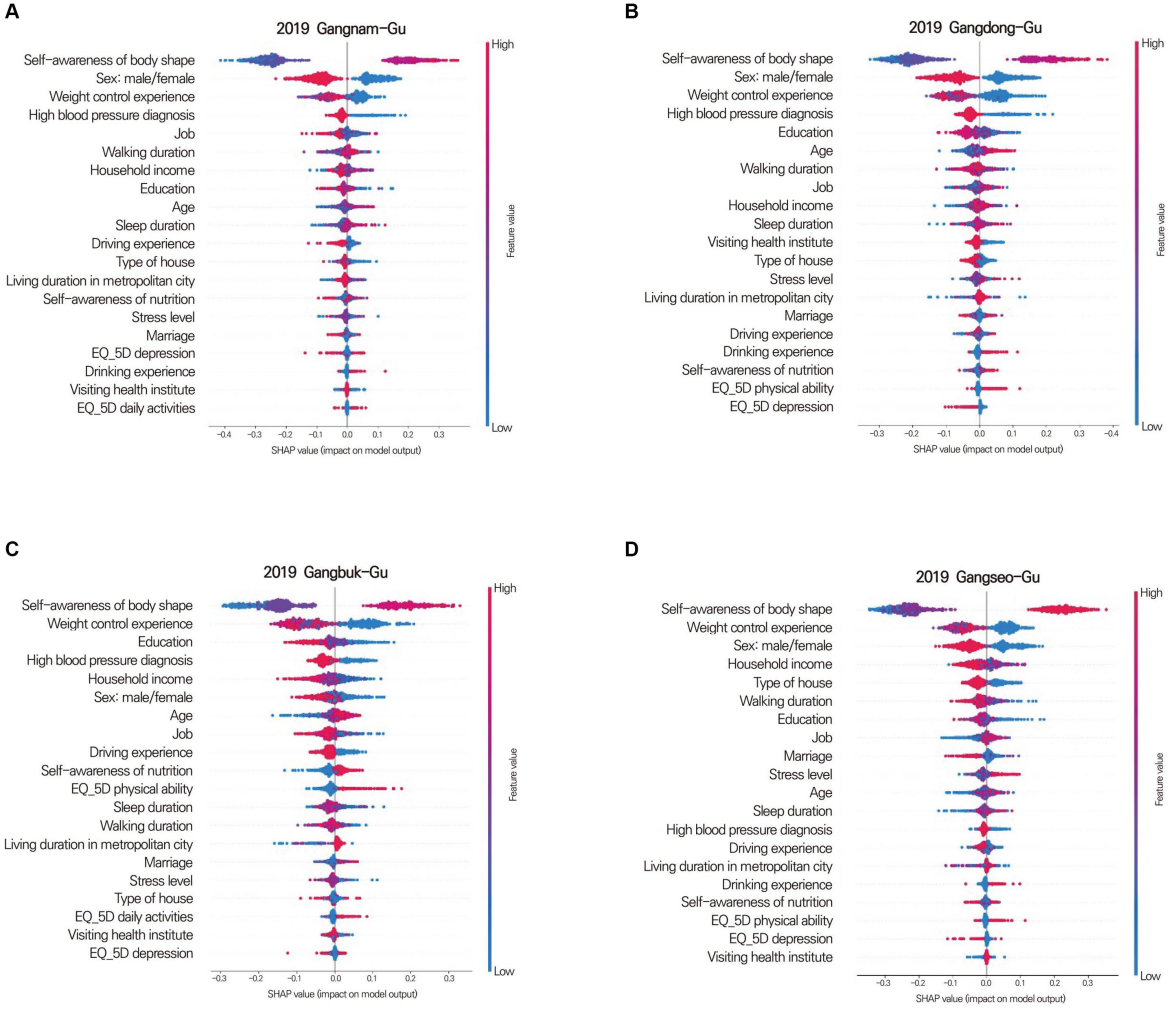
Shapley summary plot for Gangnam-Gu **(A)**, Gangdong-Gu **(B)**, Gangbuk-Gu **(C)**, and Gangseo-Gu **(D)**. The Shapley summary plot shows which factors significantly contribute to obesity, sorted in descending order of importance. For instance, in Gangnam-gu, Gangdong-gu, Gangbuk-gu, and Gangseo-gu, self-awareness of body type is the most crucial factor in determining obesity. The order of importance for each district (gu) varies in terms of the factors that contribute to obesity.

In this study, the SHAP algorithm extracted the top five features leading to obesity as individual factors. “Self-awareness of body shape” was the most influential factor in all districts. “Weight control experience” was the second most influential factor in 15 districts. Other significant influential factors were “fast food intake,” “Sex: male/female,” “high blood pressure,” “household income,” and “level of education” (Table 5; Figure 7). Although most districts had similar influential individual factors, some districts differed. In Gangseo-Gu, “house type” was an important factor leading to obesity. “Stress level,” “sleep duration,” and “smoking” were among the top five influential individual factors in Dongdaemoon-Gu, Songpa-Gu, and Jongro-Gu.

**Table 5:** Top 5 influential individual factors leading to obesity in SHAP analysis.

	1st	2nd	3rd	4th	5th
Gangnam-Gu	SAB	Fast-food	Sex	WC	HP
Gangdong-Gu	SAB	Sex	Fast-food	WC	HP
Gangbuk-Gu	SAB	WC	HP	Fast-food	Education
Gangseo-Gu	SAB	WC	Sex	House	Income
Gwanak-Gu	SAB	Fast-food	Sex	WC	HP
Gwangjin-Gu	SAB	Fast-food	Sex	WC	HP
Guro-Gu	SAB	WC	Sex	Fast-food	HP
Geumcheon-Gu	SAB	WC	Sex	Income	Education
Nowon-Gu	SAB	WC	Sex	Income	Education
Dobong-Gu	SAB	Sex	Fast-food	WC	HP
Dondaemoon-Gu	SAB	Sex	WC	HP	Stress level
Dongjak-Gu	SAB	WC	Sex	HP	Income
Mapo-Gu	SAB	WC	HP	Sex	Job
Seodaemoon-Gu	SAB	WC	Sex	HP	Income
Seocho-Gu	SAB	WC	Western-food	Sex	Smoking
Seongdong-Gu	SAB	WC	Fast-food	HP	Sex
Seongbuk-Gu	SAB	WC	Sex	HP	Age
Songpa-Gu	SAB	WC	Sex	Sleep duration	Age
Yangcheon-Gu	SAB	Fast-food	Sex	WC	Income
Youngdeungpo-Gu	SAB	WC	Sex	Age	HH
Yongsan-Gu	SAB	WC	Sex	Nutrition	HP
Eunpyung-Gu	SAB	HP	WC	Sex	Fast-food
Jongro-Gu	SAB	WC	Sex	Smoking	Chinese-food
Jung-Gu	SAB	WC	Sex	Fast-food	HP
Jungrang-Gu	SAB	WC	Sex	HP	Fast-food

SAB, self-awareness of body type; Sex, male/female; WC, weight control experience; HP, high blood pressure experience; House, type of house; HH, type of household.

### 3.4. GWLASSO for correlating social/environmental factors with spatial obesity rate

**Figure 8:**
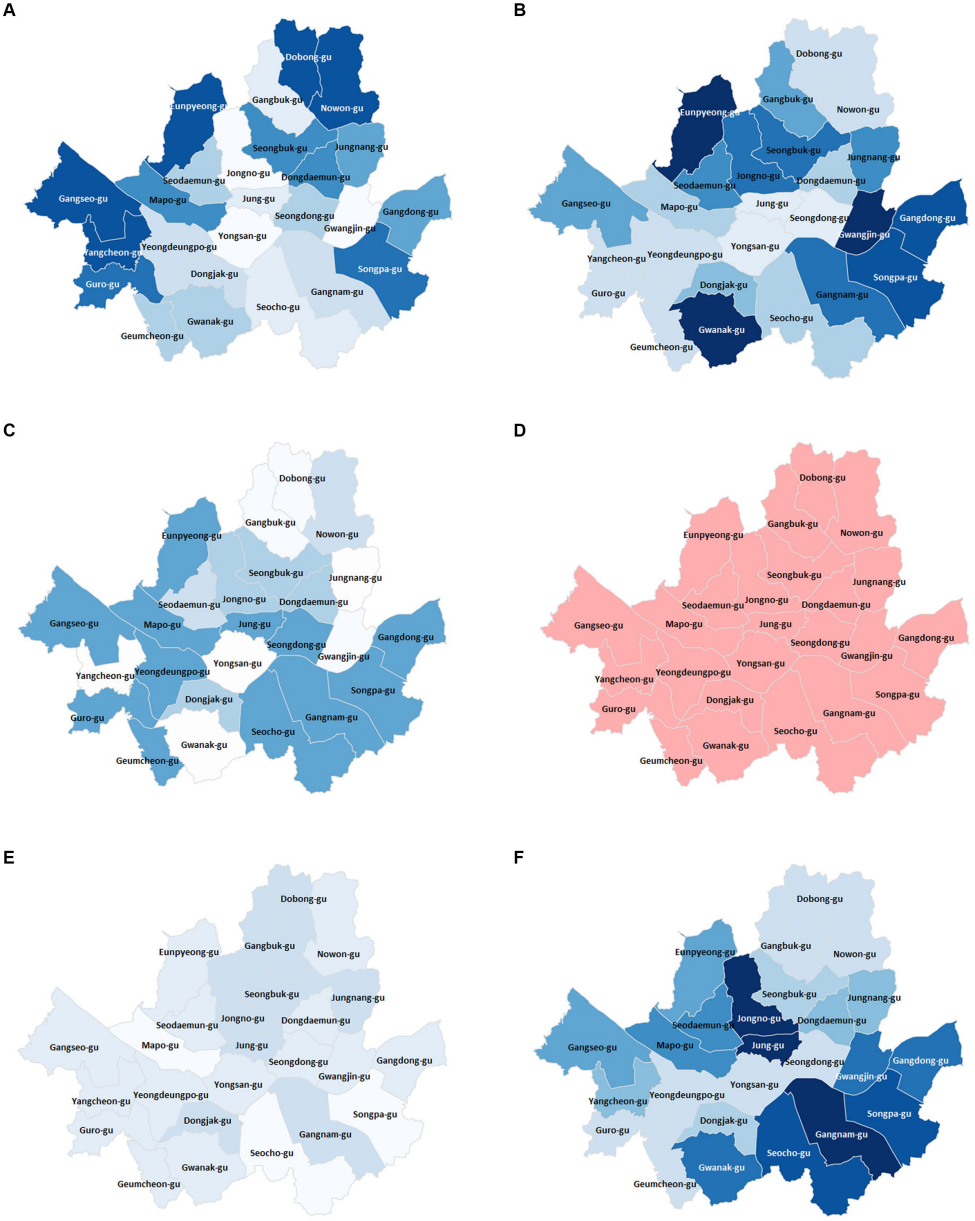
Obesity-related geographic information diagrams of geographically weighted least absolute shrinkage and selection operator for area of green park per person **(A)**, land price **(B)**, health budget **(C)**, bus utilization rate **(D)**, bus utilization rate during rush hour **(E)**, and bicycle utilization rate **(F)**. The color intensity of each district represents the correlation between obesity rate and the social/environmental factor. The stronger the color, the higher the correlation. Red and blue colors indicate negative and positive correlations, respectively. **(A)** Nowon-Gu exhibited the highest negative correlation between obesity and area of green park per person. The obesity rate in Dobong-Gu and Seongbuk-Gu, adjacent to Nowon-Gu, is also relatively highly correlated with the green park area. **(B)** All gu-districts showed decreasing obesity rates with increasing land prices. The area adjacent to Gangnam-Gu displayed a strong negative correlation between the obesity rate and land price. **(C)** Songpa-Gu had the highest negative correlation between the obesity rate and health budget. Adjacent areas such as Gandong-Gu, Gangnam-Gu, and Seocho-Gu also showed similar correlations to Songpa-Gu. **(D)** All gu-districts depicted a consistent color, reflecting that their correlation coefficients lie within the 0.60 to 0.65 range. This suggests a similar positive correlation between bus utilization rate and obesity rate across all districts. **(E)** Unlike bus utilization rate, bus utilization rate during rush hour was negatively correlated with obesity. However, the correlation coefficients were relatively small, with the highest and lowest values being −0.32 and −0.12, respectively. **(F)** The spatial correlation between bicycle utilization rate and obesity rate showed a negative relationship across all gu-districts. Among them, Gangnam-Gu exhibited the strongest negative correlation. Spatially, the neighboring areas around the district also had a similarly high negative correlation.

GWLASSO was used to derive the social/environmental factors related to obesity by considering spatial relationships. The “green park area per individual” was negatively correlated with obesity in all districts. The maximal correlation value was −0.80 in Nowon-Gu, and Seongbuk-Gu and Dobong-Gu also had a relatively strong negative correlation between the “green park area per individual” and obesity. “Land price” was negatively related to obesity in all districts. The highest correlation coefficient was −0.89 in Gwanak-Gu, and the lowest was −0.26 in Yongsan-Gu. The area around Gangnam-Gu, including Songpa-Gu and Seocho-Gu, had a relatively high negative correlation with “land prices.” “Bus utilization rate” had the opposite result from “Bus utilization rate during rush hour” and “Bicycle utilization rate” in all districts; “Bus utilization rate” was positively correlated, while “Bus utilization rate during rush hour” and “Bicycle utilization rate” were negatively correlated with obesity. The obesity rate decreased with increasing “Personal health budget” in 22 of the 25 districts, however, the coefficient was not significantly higher than other social/environmental factors (Table 6; Figure 8).

**Table 6:** The correlation coefficient between a social/environmental factor and obesity in GWLASSO.

Feature	Min	Mean	Max	POS	NEG
Area of green park per 1 person	−0.80	−0.22	−0.02	0	25
Land price	−0.89	−0.52	−0.26	0	25
Health budget	−0.42	−0.22	0.015	3	22
Bus utilization rate	0.60	0.62	0.65	25	0
Bus utilization rate in rush hour	−0.32	−0.25	−0.13	0	25
Bicycle utilization rate	−0.89	−0.56	−0.24	0	25

POS, the number of districts showing positive correlation; NEG, the number of districts showing negative correlation.

## 4. Discussion

### 4.1. Overview of study approach and findings

This study aimed to develop a machine learning model to discriminate obesity and extract significant influential individual factors using the SHAP algorithm and to derive obesity-related social/environmental factors from GWLASSO. The community health survey and credit card use data were employed to identify individual factors, while Seoul open data were used to determine social/environmental factors. Regarding individual factors, the random forest algorithm was selected by evaluating performance in the community health survey data. All individual data were used to train and validate the random forest algorithm after preprocessing to integrate the coding types, remove outliers, and normalize the data. The SHAP algorithm determined the individual-related feature importance by calculating the contribution of each feature to the prediction. Additionally, the GWLASSO identified the social/environmental factors influencing obesity by considering the spatial relationships of each district. The main findings showed that: (1) the trained model with the random forest algorithm yielded an accuracy of 90% (SD = 3%) for discriminating obesity. (2) The most influential individual factors were “weight control experience,” “fast food intake,” “Sex: male/female,” “high blood pressure experience,” “household income,” “sleep duration,” and “level of education.” (3) The GWLASSO revealed that obesity was negatively correlated with “green park area per individual,” “official land price,” “personal health budget,” “bus utilization rate during rush hour,” and “bicycle utilization rate,” in contrast to “bus utilization rate,” which positively correlated with obesity.

The random forest model trained by using survey data successfully predicted obesity with an accuracy exceeding 90%. Several studies have been performed to develop statistical and machine learning models to predict obesity. Dugan et al. developed a Naïve Bayes machine learning model that was trained and validated using clinical data (49). The accuracy of the trained model was 85%. Similar to the present study, a random forest algorithm was used to develop an obesity prediction model. The model was trained by using a dataset related to the participants’ demographic data and predicted obesity with an accuracy of 90% (69). Hammond et al. used electronic health records to develop a machine-learning model to classify childhood obesity (70). The accuracy of the model was 82% for girls and 76% for boys (70). The performance of the obesity prediction model in the present study was comparable to or better than those reported in previous studies. This suggests that a model trained by using open public data can reasonably predict obesity.

### 4.2. Individual factors identified by the SHAP algorithm

The SHAP algorithm was employed to address the non-interpretable nature of the machine learning model, revealing the elements that significantly contribute to obesity classification. Notably, the most influential individual factor for obesity classification, according to the SHAP results, was ‘self-awareness of body shape’. This finding suggests that getting individuals interested in their own body shape, rather than a behavior change, is an essential element for preventing obesity. However, it is essential to approach this recommendation with sensitivity, acknowledging the body positivity movement’s emphasis on self-acceptance and mental well-being over mere physical appearance. Media and education can play important roles in promoting a comprehensive view of health (71–73). Rather than emphasizing narrow ideals of body shape, media can focus on the broader benefits of maintaining a healthy lifestyle and the intrinsic advantages of feeling good in one’s body. Educational environments can provide learning opportunities that foster an appreciation for diverse body types and the importance of mental health. Finally, individuals could develop a balanced “self-awareness of body shape” that prioritizes a healthy body shape.

Personal experiences of weight control and high blood pressure were identified as crucial factors in the development of obesity. “Weight control experience” is related to “self-awareness of body shape.” If individuals recognize their current body shape status, they can decide whether they need to control their weight. Education on healthy body shapes may encourage overweight individuals to control their weight. “High blood pressure experience” potentially leads to “weight control experience.” Obesity is considered a high-risk factor for high blood pressure (74–76). Once individuals are diagnosed with high blood pressure, physicians usually recommend weight control. Therefore, this study’s findings suggest that education regarding body shape and regular medical checks might be associated with reduced risk of obesity development.

In the results of the SHAP algorithms, “fast-food intake” and “household income” were in the top five influential factors related to obesity in 16 of the 25 districts. Previous studies reported that the prevalence of obesity increases with decreasing household income and that household income has a significant negative relationship with fast-food intake (77–79). That is, lower income is one of the main reasons for increasing fast-food intake, which contributes to the development of obesity. To address the relationship between household income and fast-food intake, knowledge transfer for the optimization of nutritional behavior should be implemented for having healthy food regardless of income.

### 4.3. Social/environmental factors identified by GWLASSO

The GWLASSO findings suggest that, among social/environmental factors, “green park area per individual,” “bus utilization rate during rush hour,” and “bicycle utilization rate” were negatively correlated with obesity. These factors partially represent physical activity. The green park areas were related to accessibility. Bus or bicycle use induces more physical activity than the use of one’s own vehicle. Interestingly, “bus utilization rates” were positively correlated with obesity rates across all gu-districts, in contrast to “bus utilization during rush hour”. People are more compelled to use public transportation during rush hours. This might cause “bus utilization during rush hour” to have a less pronounced relationship with income. However, general bus utilization might be more closely tied to income, a significant factor for obesity. Typically, lower income correlates with higher obesity rates. These findings support a previous study in which the decreasing obesogenic environment was an important reason for the impeding obesity epidemic (45, 80, 81). Unlike individual-related factors, social/environmental factors affect most individuals around the public transportation system or healthy living spaces while minimizing the dead zone (36). Therefore, the number of public facilities, including green parks and public transportation, should be increased to prevent obesity.

The GWLASSO results demonstrated how the social/environmental determinants of obesity varied between districts. For example, the influence of “green park area per individual” on obesity ranged widely, from a negative correlation (Nowon-Gu, *r* = −0.80) to a weak positive correlation (Gangnam-Gu, *r* = 0.04). Similarly, the impact of “health budget” on obesity also varied between a negative correlation (Songpa-Gu, *r* = −0.42) and a weak positive correlation (Dobong-Gu, *r* = 0.015). In contrast, “land prices” impeded obesity across all districts. Each district had distinct characteristics. Factors with a wide range of correlations would depend on the characteristics of each district; however, the relatively consistent factor among different districts was partially independent of the characteristics of the district. Therefore, this finding suggests that how influential factors respond to the characteristics of an area should be considered when developing obesity-prevention policies or programs.

### 4.4. Combined influence of individual and social/environmental factors on obesity

Interpreting the combined influence of individual and social/environmental factors allows us to envision a comprehensive approach toward obesity prevention. For instance, the current study reveals an interaction between ‘bus utilization rate during rush hour’ as a social/environmental factor and ‘weight control experience’ as an individual factor. Increased bus utilization during rush hour, indicative of enhanced physical activity, associates negatively with obesity rates. This relationship presents a potential strategic initiative where promoting the benefits of public transportation usage and its link to physical activity could stimulate healthier behaviors. However, such an initiative necessitates a reliable public transportation system, underscoring the importance of strategic urban planning in fostering healthier lifestyles.

Unlike individual factors, social/environmental factors are not directly related to individual weight or BMI. This characteristic of social/environmental factors creates a challenge for the development of machine learning models. To address this limitation, social/environmental factors were mapped to the rate of obesity considering spatial relationships. Although GWLASSO revealed the relationship between social/environmental factors and obesity in each Gu district, it was limited in defining an accurate relationship between the rate of obesity and more detailed factors. The labeled social/environmental data accumulation would improve the ability of the model to identify the exact effects of social/environmental factors on obesity.

### 4.5. Implications for urban planning and obesity prevention strategies

In this study, we aimed to comprehensively understand the interplay between individual and social/environmental factors contributing to obesity in each district of Seoul city. By utilizing public data provided by MHOW and Seoul city, we were able to derive these contributing factors. Developing efficient and effective obesity prevention programs requires the identification of universal factors that encompass both individual and social/environmental aspects. While previous studies have reported significant individual or social/environmental obesity-related factors, integrating these results can be challenging due to the heterogeneity of each study. Our research addressed this limitation by examining both factors within the same area, thereby providing a more comprehensive understanding of obesity-related factors. This approach enables the design of targeted interventions that consider the intricate interdependencies between individual behaviors and social/environmental influences. The insights obtained by the universal factors may inform data-driven urban planning and infrastructure decisions for promoting healthier lifestyles and mitigating obesity prevalence.

### 4.6. Implications of the COVID-19 pandemic and recovery on obesity prevention

The global health crisis presented by the COVID-19 pandemic has had multifaceted impacts on public health, of which obesity is a significant concern (82–84). Previous studies have suggested that the pandemic exacerbated the pre-existing obesity trends due to various factors. First, lockdown measures and movement restrictions led to limited access to gyms, parks, and other recreational areas. The reduced access to public spaces led to a shift in behavioral patterns toward decreased physical activity (85–87). Moreover, disruptions to supply chains affected the availability and cost of fresh and nutritious foods, potentially leading to increased consumption of convenient and calorie-dense foods (88, 89). Economic uncertainties and pandemic-related stress have also been linked with unhealthy dietary choices and overeating.

While our study dealt with data from the pre-COVID era, the machine learning models and approaches developed here could be instrumental in understanding the post-pandemic obesity landscape. The SHAP algorithm and the GWLASSO can provide insights into new individual and environmental factors contributing to obesity in the pandemic. For instance, patterns of fast-food intake and household income dynamics might have further evolved during this period. Similarly, the significance of public spaces, such as green parks, has become more evident when considering how the limitations on outdoor activities affect obesity rates.

As cities adapt to the “new normal” post-pandemic, it is essential to consider these changing dynamics in obesity prevention strategies. The interplay between individual behaviors, social shifts, and environmental factors, as illustrated by our models, offers an integrated viewpoint that can inform future urban planning and health interventions. Emphasizing the importance of public spaces for physical activity, fostering resilience through community-driven initiatives, and developing awareness campaigns on healthy dietary habits tailored to specific factors are potential strategies to counteract the increased obesity epidemic.

### 4.7. Limitations and future directions of the study

Our study has successfully identified various social and environmental factors associated with obesity in Seoul City, and provided insights into how these factors interact spatially. Despite these contributions, several limitations exist within the study. First, our data was limited to datasets available up to 2019, which might not fully capture more recent trends in obesity-related factors. Factors affecting obesity rates are subject to change over time, influenced by the growth of social networking services and generational shifts in attitudes toward obesity. To devise the most efficient obesity prevention programs, it is essential to investigate risk factors using the most current resources available. Second, methodologically, the choice of machine learning algorithms and feature selection processes could have impacted the results, as different approaches might yield different outcomes. Future research should explore alternative machine learning and feature selection methods to obtain more reliable results. Lastly, the Euclidean distance used in the GWLASSO analysis might not accurately reflect the actual distance between Gu districts, because it does not account for transportation networks, topography, or other geographical barriers. In the future, analyzing data at finer spatial scales is necessary for improved accuracy.

## 5. Conclusion

This study utilized machine learning approaches to discriminate obesity and identify significant individual and social/environmental factors influencing each Gu district in Seoul by using public and open data. In addition to traditional machine-learning approaches, this study determined individual factors’ contribution to obesity using the SHAP algorithm. Furthermore, spatially dependent relationships between obesity and social/environmental factors were derived based on GWLASSO, which overcame the limitation of social/environmental factors not being labeled. This study’s findings contribute to the development of a more efficient obesity prevention program by suggesting the most significant individual and social/environmental obesity-related factors for each Gu district. This study’s findings are expected to facilitate the combination of individual-based programs with social/environment-based programs and contribute to the development of more effective and efficient obesity prevention programs.

## Data availability statement

The raw data supporting the conclusions of this article will be made available by the authors, without undue reservation.

## Author contributions

SJ: Conceptualization, Visualization, Writing – original draft. SY: Formal analysis, Investigation, Methodology, Validation, Writing – review & editing. SP: Funding acquisition, Project administration, Resources, Supervision, Writing – review & editing. SM: Conceptualization, Funding acquisition, Project administration, Resources, Supervision, Writing – review & editing.
